# Effect of Homogenization on the Transformation Temperatures and Mechanical Properties of Cu_15_Ni_35_Hf_12.5_Ti_25_Zr_12.5_ and Cu_15_Ni_35_Hf_15_Ti_20_Zr_15_ High-Entropy Shape Memory Alloys

**DOI:** 10.3390/ma16083212

**Published:** 2023-04-19

**Authors:** Shu-Yu Kuo, Wei-Pin Kao, Shan-Hsiu Chang, Ting-En Shen, Jien-Wei Yeh, Che-Wei Tsai

**Affiliations:** 1 Department of Materials Science and Engineering, National Tsing Hua University, Hsinchu 30013, Taiwan; 2 High Entropy Materials Center, National Tsing Hua University, Hsinchu 30013, Taiwan

**Keywords:** shape memory alloy, high-entropy alloy, homogenization, martensitic transformation, NiTiHfZr

## Abstract

The major challenge of high-temperature shape memory alloys (SMAs) is the collocation of phase transition temperatures (TTs: M_s_, M_f_, A_s_, A_f_) with the mechanical properties required for application. Previous research has shown that the addition of Hf and Zr into NiTi shape memory alloys (SMAs) increases TTs. Modulating the ratio of Hf and Zr can control the phase transformation temperature, and applying thermal treatments can also achieve the same goal. However, the influence of thermal treatments and precipitates on mechanical properties has not been widely discussed in previous studies. In this study, we prepared two different kinds of shape memory alloys and analyzed their phase transformation temperatures after homogenization. Homogenization successfully eliminated dendrites and inter-dendrites in the as-cast states, resulting in a reduction in the phase transformation temperatures. XRD patterns indicated the presence of B2 peaks in the as-homogenized states, demonstrating a decrease in phase transformation temperatures. Mechanical properties, such as elongation and hardness, were improved due to the uniform microstructures achieved after homogenization. Moreover, we discovered that different additions of Hf and Zr resulted in distinct properties. Alloys with lower Hf and Zr had lower phase transformation temperatures, followed by higher fracture stress and elongation.

## 1. Introduction

High-entropy alloys (HEAs) have been extensively studied by researchers since Yeh et al. pioneered the field in 2004 [1,2]. Gradually, HEAs have found applications in various fields, such as biochemistry [3], magnetics [4], and polymers [5]. Among them, high-entropy shape memory alloys (SMAs) were first developed by Firstov in 2015 [6,7]. The Ti_16.667_Zr_16.667_Hf_16.667_Ni_25_Cu_25_ alloy undergoes reversible martensitic transformation and exhibits shape memory effects. This combination of HEA and SMA has been the subject of thousands of previous studies. Due to their unique shape memory effects and super-elasticity [8,9], they have been widely investigated for their superior mechanical properties and various applications [10,11,12,13,14,15].

Homogenization heat treatment has been widely used in high-entropy alloys to improve their homogeneity [16,17,18,19]. Previous studies, such as those by Chen et al. [20,21] have investigated the effect of homogenization on shape memory effects and super-elasticity properties. The major change observed was a decrease in the four phase transformation temperatures, known as A_f_, A_s_, M_s_, and M_f_, after the heat treatment. The phase transformation temperatures are related to the crystal structures, which gradually change from the B19′ phase to the B2 phase at room temperature as the time of homogenization increases. Homogenization also eliminates dendritic structures in the matrix, while the Ti_2_Ni-like precipitates and (Ti, Zr Hf) carbides can still be observed after homogenization. Other influences of homogenization on Ti_2_Ni-like precipitates are discussed in [22], in which segregations and Ni-rich precipitations were eliminated, leading to a decrease in hardness and an increase in ductility. Research [21,23,24,25] has also pointed out that the proportion of Ti_2_Ni-like precipitation would influence the composition of the matrix phase after homogenization. They indicated that the volume fraction of the Ti_2_Ni-like phase would increase, and the size of the precipitates would decrease after heat treatment, greatly affecting the usage of such shape memory alloys. However, no study has demonstrated the mechanical compression properties or their relationships with microstructures after homogenization. Additionally, the effect of Ti_2_Ni-like homogenization on mechanical compression properties is not fully understood. Furthermore, recent research has mainly focused on the change in phase transformation resulting from homogenization without thoroughly exploring its impact on mechanical properties.

Additions of Hf and Zr, at different amounts, in shape memory alloys can result in varying phase transformation temperatures, microstructures, and mechanical properties. Previous research has highlighted the positive impact of Hf and Zr addition on shape memory alloys, enhancing strength and increasing the phase transformation temperatures [26,27,28,29,30,31]. Studies on the Ni-Ti-Hf system have also shown that the addition of of Hf and Zr at 10% can significantly increase the phase transformation temperature, with Hf having a more pronounced effect [32,33,34,35,36]. In other words, a slight change in the composition of Hf and Zr could have a significant influence on several properties, particularly with additions above 10%. Additionally, the effect of the Hf/Zr ratio on the shape memory properties of Ni_50.3_Ti_29.7_(HfZr)_20_ alloys has been studied, and it has been demonstrated that the proportion of Hf and Zr can impact the alloy’s properties [37]. However, the addition of Hf and Zr can also lead to alloy embrittlement, which can affect mechanical properties [35,38,39]. Although previous research has partially addressed the influence of Hf and Zr on the alloy’s properties, there is still a lack of research on microstructures and phase transformation temperatures with different additions of Hf and Zr. Furthermore, the effects of varying Hf and Zr compositions on the properties of shape memory alloys are not yet fully understood, and the lack of research in this area may hinder their potential applications. Therefore, further investigation is necessary to clarify the impact of different Hf and Zr additions on the microstructures, phase transformation temperatures, and mechanical properties of these alloys.

In our previous work, we successfully improved the mechanical properties and shape memory effects of the Cu_15_Ni_35_Ti_25_Hf_12.5_Zr_12.5_ shape memory alloy [40]. However, the effects of homogenization are still unclear, and no comparison of different proportions of Hf and Zr in shape memory alloys has been made. In this study, we focus on investigating the effects of varying proportions of Hf and Zr and the influence of homogenization on shape memory alloys. Microstructures will be examined and EDS analysis will be conducted to observe the changes in the elements. Thermal analysis tests will be performed to analyze the changes in the phase transformation temperatures, and XRD diffractograms will be compared to verify the results of the changes in TTs. Finally, compression tests will be used to confirm the changes in the mechanical properties.

## 2. Materials and Methods

The Cu_15_Ni_35_Hf_12.5_Ti_25_Zr_12.5_ and Cu_15_Ni_35_Hf_15_Ti_20_Zr_15_ alloy ingots, known as Cu15HZ25 and Cu15HZ30, respectively, were prepared by vacuum arc melting. Each ingot weighed 100 g, and 99.9 wt% pure elements were used. The vacuum chamber was purged at least three times to prevent oxidation during melting. Each ingot was flipped and remelted four times to ensure homogeneity. Subsequently, Cu_15_Ni_35_Hf_12.5_Ti_25_Zr_12.5_ and Cu_15_Ni_35_Hf_15_Ti_20_Zr_15_ samples were sliced into pieces and sealed in a quartz tube for homogenization at 1000 °C for 4 h, followed by air cooling. The two samples were referred to as HCu15HZ25 and HCu15HZ30 in their as-homogenized states. To prepare the test samples, they were ground with SiC sandpaper of various grits (#80, #400, #800, #1200, #2500, and #4000) until the surfaces were smooth. They were then polished with Al_2_O_3_ of 0.3 and 0.05 μm. The microstructures were examined using scanning electron microscopy (SEM, JEOL JSM-IT 100) at a voltage of 20 kV. The crystal structures were analyzed using an X-ray diffractometer (Bruker D2 Phaser X-ray) with a Cu target, and the lattice constant calculations were carried out using Rietveld refinement with the aid of Maud software due to the monoclinic crystal structure. Rietveld refinement minimized the differences in the observed data, requiring a high-quality diffraction pattern. After importing the XRD diffraction datafile and lattice constants, Maud software was used to calculated the theoretical spectrum and refine the true constant lattice values. If there was a discrepancy between the constant lattice values obtained by Maud and the lattice values calculated using XRD data, several equations could be used to determine which one is correct.

(1) $$\frac{1}{d^{2}}=\frac{1}{\sin^{2}\beta }\left(\frac{h^{2}}{a^{2}}+\frac{k^{2}\sin^{2}\beta }{b^{2}}+\frac{l^{2}}{c^{2}}-\frac{2\mathrm{hlcos}\beta }{ac}\right),$$

(2) nλ=2dsinθ,

To verify the accuracy of the fitting results obtained from Maud, the lattice constants calculated by Maud and the corresponding Miller indices were inputted into the equations mentioned above to obtain θ values. These θ values were than compared to the θ values of the XRD peaks.

The samples were prepared for thermal cycling tests by shaping them into cylinders, 6 mm in diameter and 6 mm in height. The dilatometer (DIL, NETZSH 403 E select) was used to analyze the samples at a heating and cooling rate of 3 °C/min. Differential scanning calorimetry (DSC, NETZSH 204 F1 LT-DSC) was also employed to verify the phase transformation temperatures at a heating and cooling rate of 3 °C/min. For compression tests, the samples were sliced into cylindrical shapes, 5 mm in diameter and 6 mm in height. A universal testing machine (Instron 4468) was used to analyze the samples at a strain rate of $10^{-3}\;s^{-1}$.

## 3. Results

### 3.1. Microstructures

**Figure 1:**
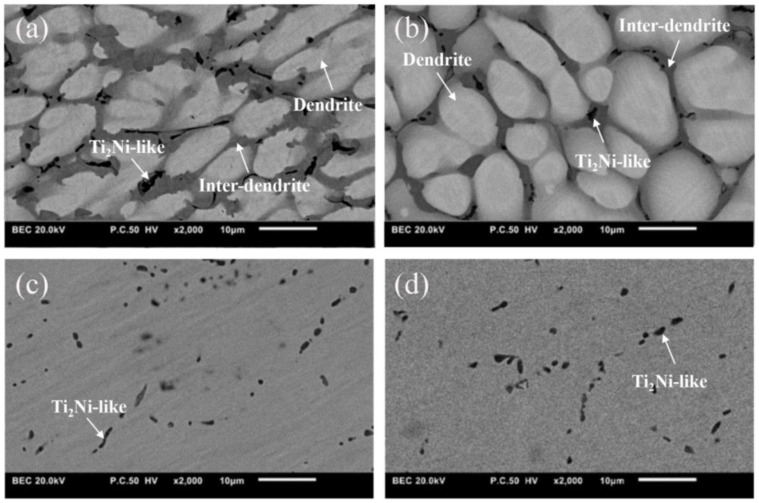
Microstructure of as-cast states (**a**) Cu15HZ25 and (**b**) Cu15HZ30; microstructure of as- homogenized states (**c**) HCu15HZ25 and (**d**) HCu15HZ30.

Figure 1a,b display the backscattering electron (BSE) images of the as-cast samples of Cu15HZ25 and Cu15HZ30, respectively. In these images, the microstructures of both alloys appear dendritic, with visible segregation, and black precipitates can be observed within the matrix. The lighter areas correspond to dendrites, while the darker areas belong to the inter-dendrite region. Figure 1c,d show the microstructures of HCu15HZ25 and HCu15HZ30 after homogenization. In these images, the dendritic structures have been successfully eliminated, leaving a gray matrix with black precipitates in the microstructure. The homogenization process also led to a significant reduction in the segregation that was present in the as-cast states. Table 1 provides the composition of the as-cast and as-homogenized states. The data reveal that the composition of the matrix was close to the nominal composition of the alloy after homogenization. EDS examination indicated that the composition of the precipitates was similar to (TiHfZr)_2_(CuNi), also known as the Ti_2_Ni-like phase in previous studies [20]. The volume fraction of the Ti_2_Ni-like precipitates increased after homogenization for both alloys, with Cu15HZ25 increasing from 3.13% to 3.49% and Cu15HZ30 increasing from 1.93% to 2.23%. Additionally, due to the increase in Ti_2_Ni-like precipitates, the (Ti + Zr + Hf) content of the matrix also increased slightly after homogenization.

### 3.2. X-ray Curves

**Figure 2:**
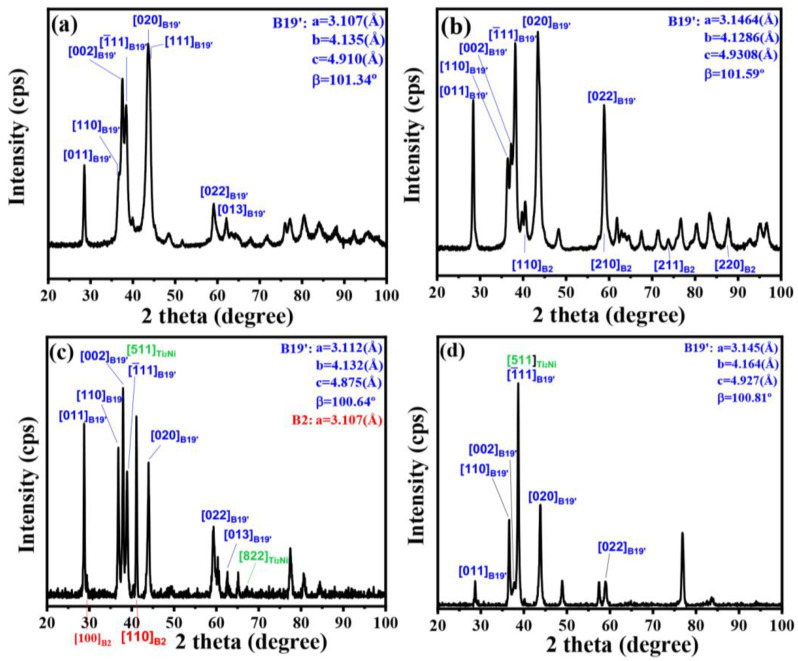
XRD diffractograms of (**a**) Cu15HZ25, (**b**) Cu15HZ30, (**c**) HCu15HZ25, and (**d**) HCu15HZ30.

Figure 2 presents the diffraction patterns of diffractograms of Cu15HZ25 and Cu15HZ30 both in as-cast and as-homogenized states, as depicted in Figure 2a, b, respectively. These patterns indicate that the B19′ martensite is the primary constituent of both alloys at room temperature in the as-cast state. In Cu15HZ30, the lattice constants of B19′ were found to be a = 3.146 (Å), b = 4.129 (Å), c = 4.931 (Å), and β = $101.59^{{}^{\circ}}$, with a calculated volume of 62.749 (${Å}^{3}$) by V=a×b×c×sinβ. Although a small amount of B2 austenite was observed in Cu15HZ30, B19′ martensite remained the dominant structure. Similarly, the lattice constants of Cu15HZ25′s B19′ were a = 3.107 (Å), b = 4.135 (Å), c = 4.910 (Å), and β  = $\;101.34^{{}^{\circ}}$, with a calculated volume of 61.856 (${Å}^{3}$) for B19′ unit cells. After homogenization, both HCu15HZ25 and HCu15HZ30 still consisted mainly of B19′ martensite at room temperatu re, as shown in Figure 2. The lattice structure of HCu15HZ25 was found to be monoclinic with a P21/m space group, and the lattice constants of B19′ were determined to be a = 3.112 (Å), b = 4.132 (Å), c = 4.875 (Å), and β = $100.64^{{}^{\circ}}$. In HCu15HZ25, a small amount of B2 austenite coexisted with B19′ martensite at room temperature. The B2 austenite was found to belong to BCC structures, with a lattice constant of a = 3.107 (Å). The lattice structure of HCu15HZ30 was the same as that of HCu15HZ25, with lattice constants of B19′ being a = 3.145 (Å), b = 4.164 (Å), c = 4.927 (Å), and β = 100.81. After homogenization, Ti_2_Ni-like peaks were also observed in both alloys, and were consistent with the results of the microstructure and EDS analyses.

### 3.3. Phase Transformation Temperatures of M_s_, M_f_, A_s_, and A_f_

**Figure 3:**
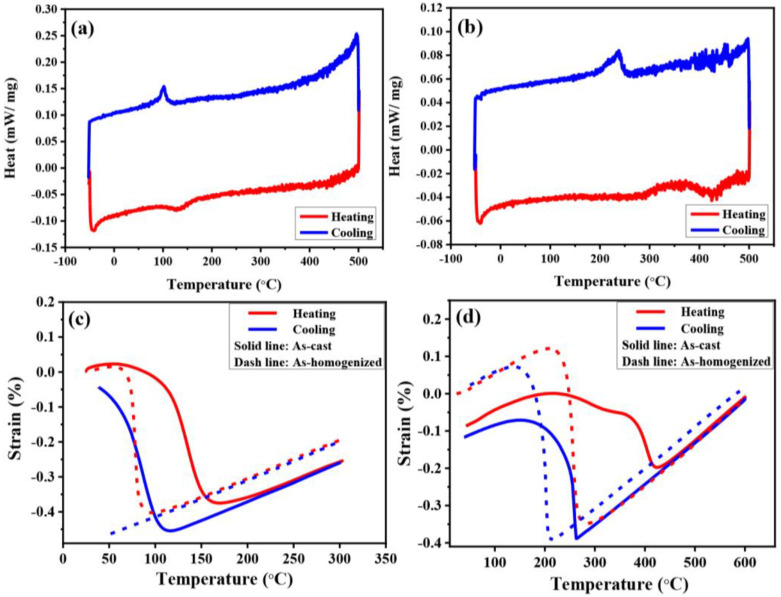
Thermal analysis of as-cast and as-homogenized states. (**a**) Cu15HZ25 and (**b**) Cu15HZ30, analyzed by DSC; (**c**) Cu15HZ25 and (**d**) Cu15HZ30, analyzed by DIL.

The DIL and DSC tests revealed that the phase transformation temperatures of the shape memory alloys Cu15HZ25 and Cu15HZ30 were influenced by the proportion of Hf and Zr. Higher proportions of Hf and Zr resulted in higher phase transformation temperatures and larger hysteresis loop areas, as shown in Figure 3 and Table 2. The thermal hysteresis area of Cu15HZ30, represented by ΔT (equal to the value of A_f_-M_s_), sharply decreased from 160 to 59 after homogenization, as shown in Figure 3d. In the as-cast state, the A_f_ values of Cu15HZ25 and Cu15HZ30 were 164 °C and 424 °C, respectively. After homogenization, there was a significant decrease in all four phase transformation temperatures. For Cu15HZ25, the A_f_ decreased from 164 to 83 °C, whereas for Cu15HZ30, it decreased from 424 to 265 °C. However, due to temperature limitations, DIL did not observe the M_f_ or M_s_ of Cu15HZ25 due to temperature limitations, and the phase transformation temperatures were examined by DSC instead. The A_s_ and A_f_ of Cu15HZ25 were 95.6 and 169.3 °C. The A_f_, A_s_, M_s_, and M_f_ of Cu15HZ30 were 325.0, 248.6, 252.3, and 192.2 °C, respectively. After homogenization, the phase transformation temperatures of both alloys decreased, regardless of the different Hf and Zr ratios. There were slight differences in the temperatures obtained from DIL and DSC, which might be due to experimental variations and measuring disparities between different experimental tools.

### 3.4. Mechanical Properties

**Figure 4:**
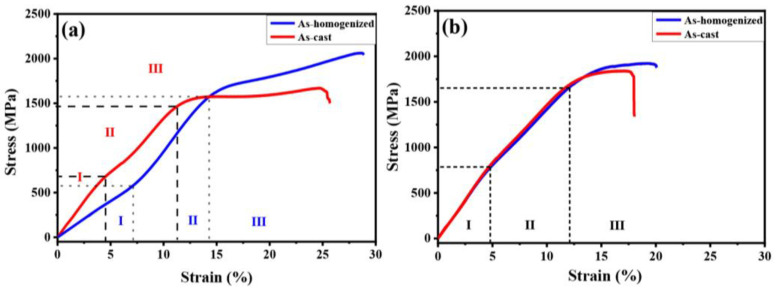
Compressive stress–strain curves of (**a**) Cu15HZ25 and (**b**) Cu15HZ30 in as-cast and as-homogenized states.

The compression curves presented in Figure 4 demonstrate that the stress–strain relationship of the compression tests can be divided into three distinct regions. In region I, the stress increased slowly due to elastic deformation, followed by a small amount of plastic deformation. All curves exhibited a slow increase in stress in this stage, with stress values lower than 750 MPa. In region II, the stress–strain curve became nearly linear, but with a different slope than in region I, indicating that the alloy underwent strain hardening throughout the compression test. This stage was characterized by relatively unapparent dislocations and a detwinning effect [41,42]. During this stage, the variants in the martensite phase gradually changed directions, which is referred to as the detwinning progress. Theoretically, this stage should have presented a plateau region, but the proliferation of dislocations in the microstructure during the compression test caused the curve to appear linear. Stress values in region II ranged between 750 and 1500 MPa. In region III, the stress–strain curve demonstrated obvious plastic deformation, generating severe slips and a significant number of dislocations until the alloy ultimately fractured. These results are consistent with those reported in [37].

Table 3 shows a comparison of the mechanical properties of the alloy before and after homogenization, including fracture stress, hardness, and elongation. For Cu15HZ25, the fracture stress increased from 1667 MPa to 2061 MPa after homogenization, and the compressive elongation increased from 25.0% to 28.6%. In the case of Cu15HZ30, the fracture stress increased from 1836 MPa to 1902 MPa, and the compressive elongation increased from 17.5% to 20.0%. However, the hardness of Cu15HZ25 decreased from 317.7 ± 3.7 to 264.6 ± 3.83.49 Hv after homogenization, and for Cu15HZ30, it decreased from 352.1 ± 8.6 to 333.6 ± 4.5 Hv. It was apparent that both Cu15HZ25 and Cu15HZ30 exhibited a significant increase in fracture stress and elongation after homogenization, as observed in Figure 4.

## 4. Discussion

Upon comparing the XRD patterns of Cu15HZ25 and Cu15HZ30, it could be noted that the lattice constants of Cu15HZ30 were consistently larger than those of Cu15HZ25, irrespective of whether they were in as-cast or as-homogenized states. It is essential to highlight that different Hf and Zr ratios did not alter the crystal structures of the alloys. However, the larger atomic radii of Hf (1.580 (Å)) and Zr (1.602 (Å)) compared to that of Ti (1.462 (Å)) led to the replacement of Ti by Hf and Zr, resulting in an increase in the unit cell volume and lattice constants. As more Hf and Zr were added, the lattice constants a, c, and β eventually decreased, although b did not exhibit any noticeable trend. The difference in Hf and Zr addition did not cause significant differences in the phase components between the two different compositions. However, after homogenization, some B2 austenite peaks appeared in HCu15HZ25 due to the decrease in phase transformation temperature, resulting in a higher probability of austenite formation at lower temperature regions. Nonetheless, no B2 peaks were observed in HCu15HZ30, indicating that the phase transformation temperature after homogenization was still not low enough to induce B2 austenite formation at room temperature.

Previous studies have demonstrated that phase transformation results in volume changes due to differences in lattice constants and crystal structures between the austenite and martensite phases. This volume change can be used to examine the respective phase transformation temperatures [43]. In addition, the homogenization of HCu15HZ30 eliminated the composition segregation of dendrites and inter-dendritic regions, which led to a significant reduction in the thermal hysteresis loop and a narrower region in the range of phase transformation. The phase compositions of Hf and Zr have been reported to strongly influence phase transformation temperatures [37,44,45,46]. The addition of Hf and Zr increases phase transformation temperatures by replacing Ti with Hf and Zr [47]. Our work clearly shows that phase transformation temperatures are consistently higher when Hf and Zr are added at 30%. Consistent with previous findings [7,20,21], we observed a decrease in phase transformation temperatures after homogenization. We discovered that the Ti_2_Ni-like volume fraction increased after homogenization, and the high proportion of Ti and Zr in this phase resulted in a decrease in phase transformation temperatures. It was observed that the Ti_2_Ni-like volume fraction, which is abundant in Ti and Zr, increased after homogenization. The grain sizes of the Ti_2_Ni-like precipitates also increased after homogenization, which primarily contributed to the decrease in the phase transformation temperature. However, the significant decrease in phase transformation temperature observed in our study cannot be solely attributed to the elimination of dendritic regions. While the elimination of dendritic structure after homogenization could possibly have led to a change in the phase transformation temperature, the dendritic and metallic area between dendrites contributed to the phase transformation of the as-cast state, and different element additions led to different amplitudes of change in phase transformation temperatures [47]. Moreover, the element segregation observed in the as-cast alloy was primarily due to changes in its composition, which should not change significantly after homogenization. Therefore, the significant change in phase transition temperature should not solely be attributed to the contribution of dendritic areas, but rather to the entire alloy. It is likely that the gradual increase in the thermal stability of the phase transformation temperature and the decrease in the phase transformation temperature were due to the gradual increase in the thermal stability of phase transformation after homogenization. In summary, the phase transformation temperatures of HCu15HZ25 and HCu15HZ30 can be influenced by various factors, including the phase compositions, volume changes, elimination of dendrite and inter-dendrite structures, and proportions of Ti, Zr, and Hf. It is important to consider all of these factors when discussing changes in phase transformation temperatures after homogenization.

Figure 4 shows the compressive stress–strain curves of Cu15HZ25 and Cu15HZ30 in both as-cast and as-homogenized states. It is evident that the compressive stress was affected by the composition of Hf and Zr. When the Hf and Zr content was lower, the compressive stress was also lower, and the compressive elongation was better. Previous studies have also discussed the mechanical properties of alloys with different ratios of Zr [48]. As the Zr content increases, the Young’s modulus and elongation decrease due to the residual stress-induced martensite of the B19′ phase that remains in the alloy under high stress conditions. Moreover, adding more Zr to NiTi SMAs can reduce processability and increase brittleness, as mentioned in [30], which is consistent with our findings. During compression tests, the compression energy results from the processes of austenite elastic deformation, stress-induced martensite transformation, and comprehensive deformation of martensite [49,50]. Differences in these three deformation conditions between as-cast and as-homogenized states can affect the mechanical properties. The ductility of shape memory alloys after homogenization has also been discussed [40,51,52], indicating that elongation increases after solution treatment. After homogenization, the compressive elongation of HCu15HZ25 and HCu15HZ30 also increase. Table 1 demonstrates that the Ti_2_Ni-like precipitate increased after homogenization. Since Ti_2_Ni-like precipitates belong to hard and brittle phases [53,54], they have a negative influence on the mechanical properties. The increase in elongation observed in our study was not influenced by the change in composition, but by the elimination of segregation in the as-cast states after homogenization. The present homogenization results led to improved compressive stress and elongation and decreased the hardness of the alloy. These results also demonstrate that homogenization achieves the goal of eliminating segregation in the as-cast states.

## 5. Conclusions

Our work provides valuable insights into the effects of homogenization on the microstructures, phase transformation temperatures, and mechanical properties of CuNiTiZrHf shape memory alloys. Through homogenization, we successfully eliminated the dendritic structure, and despite an increase in Ti_2_Ni-like precipitations, the alloy’s crystallization peaks were not significantly affected. Homogenization caused a significant reduction in the phase transformation temperature, which could be attributed to the contribution of dendrites in the surrounding areas, as well as the thermal stability of the alloy itself. Additionally, the ratio of Hf and Zr addition influenced phase transformation temperatures, with higher ratios resulting in higher phase transformation temperatures. The mechanical properties of alloys with lower ratios were characterized by higher elongation and compressive stress, but lower hardness. Moreover, homogenization improved the mechanical properties of the alloy by increasing the compressive elongation. These findings may have important implications for the development and application of CuNiTiZrHf shape memory alloys.

## Figures and Tables

**Table 1:** EDS analysis of Cu15HZ25 and Cu15HZ30 in as-cast and as-homogenized states.

	Phase	Cu	Ni	Hf	Ti	Zr	V_f_ of Ti_2_Ni-Like (%)
HCu15HZ25	Matrix	15.9	34.2	24.8	13.0	12.1	3.49%
	Precipitates	11.2	29.1	35.9	9.7	14.1	
Cu15HZ25	Dendrite	12.6	36.9	14.1	24.2	12.2	3.13%
	Interdendrite	19.6	30.1	9.8	25.3	15.2	
	Precipitates	15.7	25.2	7.9	33.6	17.6	
HCu15HZ30	Matrix	16.0	34.9	19.6	14.8	14.7	2.23%
	Precipitates	8.8	27.3	34.7	10.9	18.3	
Cu15HZ30	Dendrite	12.4	37.2	18.8	17.4	14.2	1.93%
	Interdendrite	23.9	23.5	8.0	23.3	21.3	
	Precipitates	12.3	22.3	6.4	40.3	18.7	

**Table 2:** Phase transformation temperatures for Cu15HZ25 and Cu15HZ30 in as-cast and as-homogenized states.

		M_s_	M_f_	A_s_	A_f_	ΔT
Cu15HZ25	As-cast (DSC)	111.6	99.7	95.6	169.3	61.4
	As-cast (DIL)	114	n/a	101	164	50
	As-homogenized	n/a	n/a	74	83	n/a
Cu15HZ30	As-cast (DSC)	252.3	192.2	248.6	325.0	n/a
	As-cast (DIL)	264	252	384	424	160
	As-homogenized	206	190	242	265	59

**Table 3:** Mechanical properties of as-cast and as-homogenized states of Cu15HZ25 and Cu15HZ30.

		Facture Stress (MPa)	Hardness (Hv)	Elongation (%)
Cu15HZ25	as-cast	1667 MPa	317.7 ± 3.7	25.0%
	as-homogenized	2061 MPa	264.6 ± 3.8	28.6%
Cu15HZ30	as-cast	1836 MPa	352.1 ± 8.6	17.5%
	as-homogenized	1902 MPa	333.6 ± 4.5	20.0%

## Data Availability

The data presented in this study are available upon request from the corresponding author, and data are contained within the article.
